# Welding-induced corrosion and protective measures for clad rebars in neutral chloride environments

**DOI:** 10.1038/s41598-024-56348-z

**Published:** 2024-05-22

**Authors:** Zecheng Zhuang, Weiping Lu, Lei Zeng, Jianping Tan, Xuehai Qian, Zhen Li, Wei Jiang, Yong Xiang

**Affiliations:** 1 Light Alloys Research Institute, Central South University, Changsha, 410083 China; 2 Guangxi Normal University of Science and Technology, LaiBin, 546199 China; 3 State Key Laboratory of Precision Manufacturing for Extreme Service Performance, Central South University, Changsha, 410083 China; 4 School of Mechanical and Electrical Engineering, Central South University, Changsha, 410083 China; 5 Technology Centre, Guangxi Liuzhou Iron and Steel Group Ltd., Liuzhou, 545002 China

**Keywords:** Clad rebars welding (CRW), Electrochemical corrosion, Dynamic recrystallization (DRX), Neutral chloride environment, Engineering, Materials science

## Abstract

Corrosion-resistant steel plays a vital role in marine steel structures. This study developed an SS304/HRB400 stainless-steel-clad rebar for application in a cross-sea bridge in Zhejiang Province. CO_2_ gas shielded welding was employed in the prefabricated steel structure, with SS304 steel as the welding wire. This study investigated the welding on the corrosion resistance of clad rebars and explored corrosion protection measures for welded joints.The results indicated that refined grains appeared in both stainless steel and carbon steel due to distinct dynamic recrystallization (DRX) during welding. The corrosion resistance, as determined by potentiodynamic polarization curve analysis of the material’s interaction with the solution ranked as follows: clad rebar (polished) > clad rebar welding (CRW) > painting the clad rebar after welding (PCRW) > clad rebar (unpolished) > carbon-steel welding (CSW) > carbon-steel bar > cold spraying zinc after clad rebar welding (ZCRW). However, an accelerated corrosion test with four samples for 600 s with a corrosion current of 0.8 A revealed minimal corrosion damage on zinc-coated surfaces. Hence, welding joints for clad steel structures are considered feasible and must be subject to cold zinc spraying after polishing to enhance their corrosion resistance.

## Introduction

Corrosion-resistant steel stands as a pivotal component within marine structural^1–3^. The annual corrosion-related losses in China amount to approximately 2.1 trillion yuan, with 32% attributed to corrosion of metal structures^4–6^. Developing innovative corrosion-resistant steel is paramount for marine structures. Researchers have successfully fabricated a hot-rolled SS304/HRB400 stainless-steel-clad rebar, which found application in a Zhejiang Province cross-sea bridge. This area is in the tidal range and splash area of sea water, and the corrosion is the most serious. Therefore, it is particularly important to study the corrosion resistance and protection of welded joints of clad steel structures, and provide theoretical support and scientific basis for the structural integrity evaluation and residual life prediction.

Numerous scholars globally have investigated the corrosion resistance of welded joints across various materials. Wang et al.^7^ conducted a study on SS304 and Q235 mild steel friction stir welding. Their research delved into microstructure, interface characteristics, residual stress distribution, and mechanical properties of welded joints. They examined the primary mechanisms influencing grain refinement and recrystallization in the stirring zone and thermal influence zone. Cui et al.^8^ focused on S32101 duplex stainless steel, empoying K-TIG welding within a heat input range of 1.99–2.46 kJ /mm. EBSD technology aided in analyzing and calibrating lattice grain boundaries and chromium nitridation in the weld metal. The study evaluated the mechanical properties and intergranular corrosion performance of the welded material, emphasizing the impact of chromium nitride precipitation and microstructure on alloy mechanical properties. Wei et al.^9^ conducted HIG welding on 7N01-T4 aluminum alloy plates with ER5087 and ER5356 welding wires, examining electrochemical corrosion in NaCl solution of concentrations 3.5% and 5% respectively. They demonstrate that the corrosion resistance of ER5356 welded joints in the weld zone was better than that of ER5087 welded joints. With the increase in NaCl solution concentration, the size of ER5356WM corrosion pit increased abruptly, and the corrosion mechanism transitioned from pitting corrosion to intergranular corrosion. Sohail et al.^10^ carried out the tests to evaluate the corrosion performance of mild steel, high strength steel, epoxy-coated steel and high chromium steel under harsh environment. The analysis indicates that the corrosion resistance of the evaluated steels was in the decreasing order: epoxy-coated steel, high chromium steel, mild steel, high strength steel. Shi et al.^11^ employed DP-TIG to weld 10.8 mm thick S32101 duplex stainless-steel plates,assessing intergranular corrosion resistance through electrochemical tests. The study revealed that the volume fraction of ferrite, austenite, and the orientation deviation angle of grain boundaries influence intergranular corrosion in welded joints. With increasing heat input, the austenite volume fraction and the boundary frequency of ∑3 coincident lattice increased, With the increase of the volume fraction of austenite, the corrosion resistance increases, and with the increase of ∑3 coincident lattice, the intergranular corrosion can be effectively prevented. Therefore, the higher the proportion of ∑3 coincident lattice is, the better the corrosion resistance will be. Bore et al.^12^ investigated the impact of electrochemical corrosion tests on the pitting and intergranular corrosion resistance of X5CrNi18-10 stainless-steel welded joints under different TIG welding currents. Their results indicated a decrease in pitting resistance and intergranular corrosion resistance with increased welding current. Notably, the present of nitrogen in the protective gas enhanced pitting resistance. Yang et al.^13^ employed TIG welding for ADC12 aluminum alloy and conducted electrochemical tests to examine the corrosion resistance of welded joints in various regions. The findings revealed that initially, the corrosion resistance of the welded joint surpassed that of ACD12 aluminum alloy like due to grain refinement and uniform distribution. Subsequently, the corrosion resistance of the base metal improved. Pitting corrosion emerged as the primary corrosion form, with the welded joint experiencing uniform corrosion. Senthilkumar et al.^14^ investigated ASTM A36 steel plate, exploring the impacts of different heat inputs (0.608 kJ/mm, 0.9 kJ/mm and 1.466 kJ/mm) on welding microstructure, corrosion, and mechanical properties. It was observed that as heat input increased tensile strength and hardness of the weld decreased, while the corrosion rate gradually declined. Li et al.^15^ focused on high-nitrogen austenitic stainless-steel welded joints, subjecting them to an intergranular corrosion test with multi-strand wound welding wire. The results indicated that under conditions of high heat input and low cooling rates, the DOS value of the welded joint was highest, leading to increased intergranular corrosion. Conversely, under low heat input and low cooling rates, the welded joint exhibited high corrosion resistance and limited electrochemical reactions. Gucwa et al.^16^ analyzed the corrosion resistance of SS304 welded joints under different material heat input. It was found that the microstructure and properties of austenitic stainless steel joints are largely dependent on the heat input of the weld material, and the corrosion resistance of the welded joints is worse than that of the base material. Sriba et al.^17^ utilized the GTAW welding process to joint SS316L with filler metals ER316LN and ER308LN, respectively, investigating the influence of chemical composition on the corrosion resistance of welded joints. Results revealed that the welded joint, composed of the base material, served as the anode zone, with the fusion zone acting as the cathode part. While the pitting resistance of the 316L/ER308LN welded joint exceeded that of the 316L/ER316LN counterpart the overall corrosion resistance was diminished. Li et al.^18^ explored the galvanic corrosion behavior of low carbon ferritic stainless-steel resistance welding (ERW) joints in a simulated seawater environment. The study identified typical local corrosion in the base material and welded joint area, with corrosion resistance ranked as follows: weld material > Base material > Low-temperature heat-affected zone (HAZ) > In the high-temperature HAZ. A characteristic groove corrosion pattern manifested in the joint area, with the corrosion crack initiation point identified as the HAZ, and the degree of corrosion being lighter at the weld position. However, the previous studies did not investigate the impact of welding on the corrosion resistance of the overall steel structure, nor did it study the feasibility of its welding connection mode.

In summary, in view of the fact that the clad steel structure serves in the most corrosion-prone area of the cross-sea bridge, the stainless steel clad steel structure should have good corrosion resistance. CO_2_ gas protection welding is currently proposed to connect the prefabricated steel structure. Herein, the feasibility of welding to connect the clad steel structure will be demonstrated. The influence of welding on the corrosion resistance of steel structure was studied, and whether the corrosion resistance of welded joints of clad steel meets the corrosion resistance of steel standards was judged, and a reasonable technical scheme or reasonable suggestion for the connection of prefabricated steel structure was given.

## Materials and methods

### Hot- rolling clad rebar

#### Clean interface billet process

**Figure 1:**
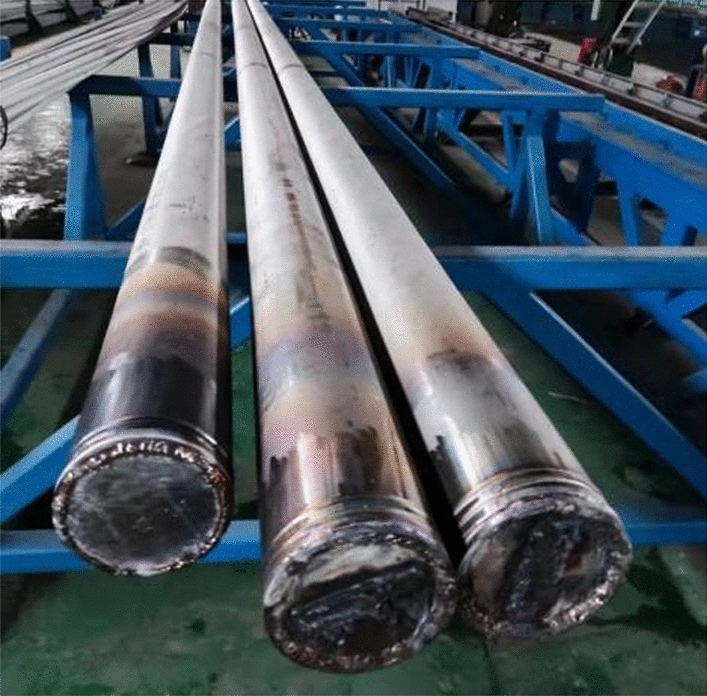
Composite billet formation process.

To make a good metallurgical combination of the stainless steel cladding and the carbon steel base metal, to remove rust and clean the outer surface of the carbon steel core rod and the inner surface of the stainless steel tube. Use acetone to clean the billet surface. The size of the stainless steel pipe is: ϕ 168 mm × 8 mm × 9000 mm, and the material of the carbon steel core rod is: ϕ 150 mm × 9000 mm. The billet was vacuumed and the end face was welded to complete the assembly of the composite billet, as shown in the Fig. 1.

#### Hot-rolling process

**Figure 2:**
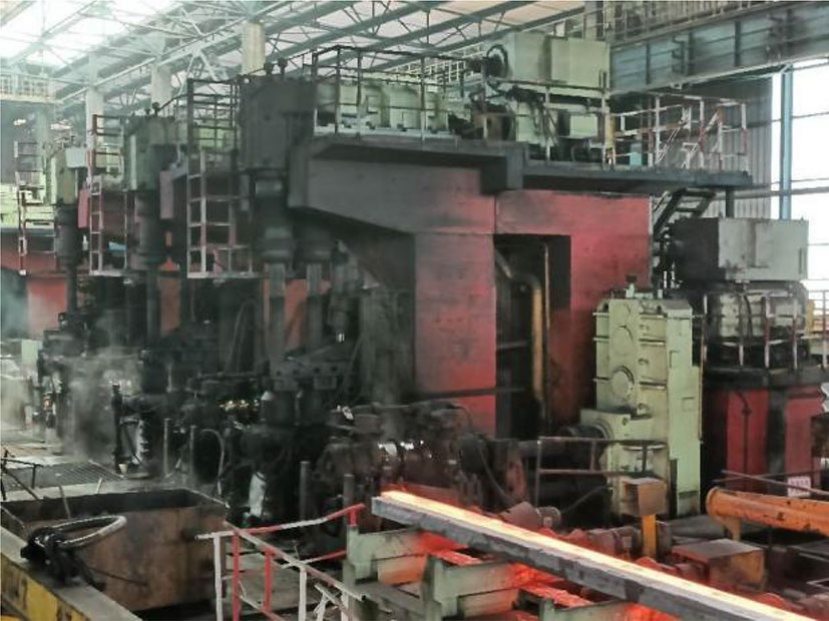
Rolling process of clad steel.

**Figure 3:**
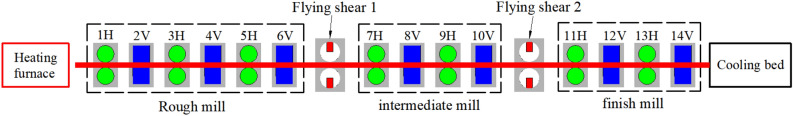
Schematic diagram of the relative position of the rolling mills and the spray point^19^.

The rolling place was in a steel plant in Guangxi, the outside diameter of the billet was 168 mm, and the rolling line used 6 rough rolling, 4 medium rolling and 4 fine rolling passes, respectively, for a total of 14 passes. The size of the finished steel bar was 16 mm. The billet rolling diagram and the rolling line layout diagram were shown in Figs. 2 and 3.

Since the composite interface of the billet is in a vacuum state, in order to correct the composite billet to be heated evenly, the heating temperature of the heating furnace is set as: 1060 °C ± 30 °C, the opening rolling temperature is about: 1090 °C, the middle rolling temperature is about: 1070 °C, and the upper cooling bed temperature is about: 1030 °C. After the rolling is finished, the covered steel bar is air-cooled to room temperature.

### Welding sample and process

**Figure 4:**
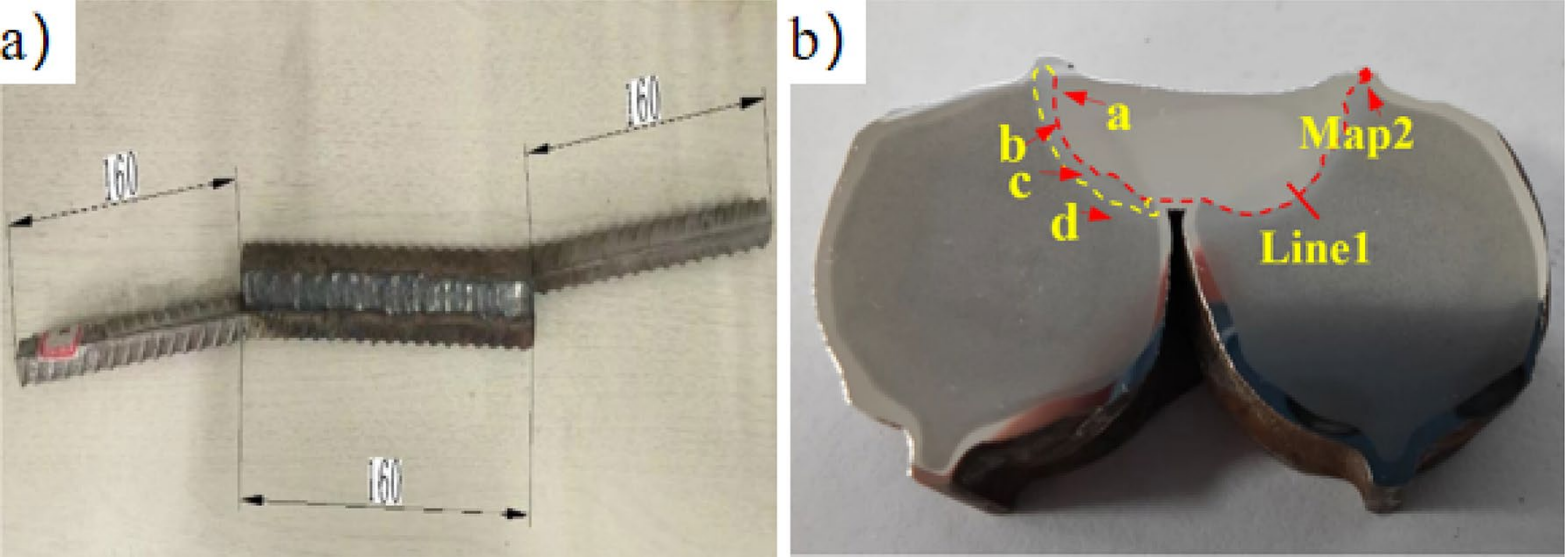
Full welded sample size on the construction site (mm): (**a**) stainless-steel-clad rebar welding sample and (**b**) metallographic, electron probe, and electrochemical samples.

The specimen was sourced from an ongoing bridge construction in Taizhou, Zhejiang Province. Comprising SS304 cladding and an HRB400 carbon-steel matrix, the clad rebar is hot-rolled with a diameter of 16 mm, a length of 320 mm per piece, a 14° bend, and a welding length of 16 mm. Welding parameters include a voltage of 70 V, SS304 welding wire, and a welding speed of approximately 16.7 mm/min. Figure 4a illustrated the welding sample of the stainless-steel-clad rebar. For subsequent tests, a 16 mm segment was extracted for metallographic, electron probe and electrochemical analyses, revealing the cross-section depicted in Fig. 4b.

### Sample preparation

**Figure 5:**
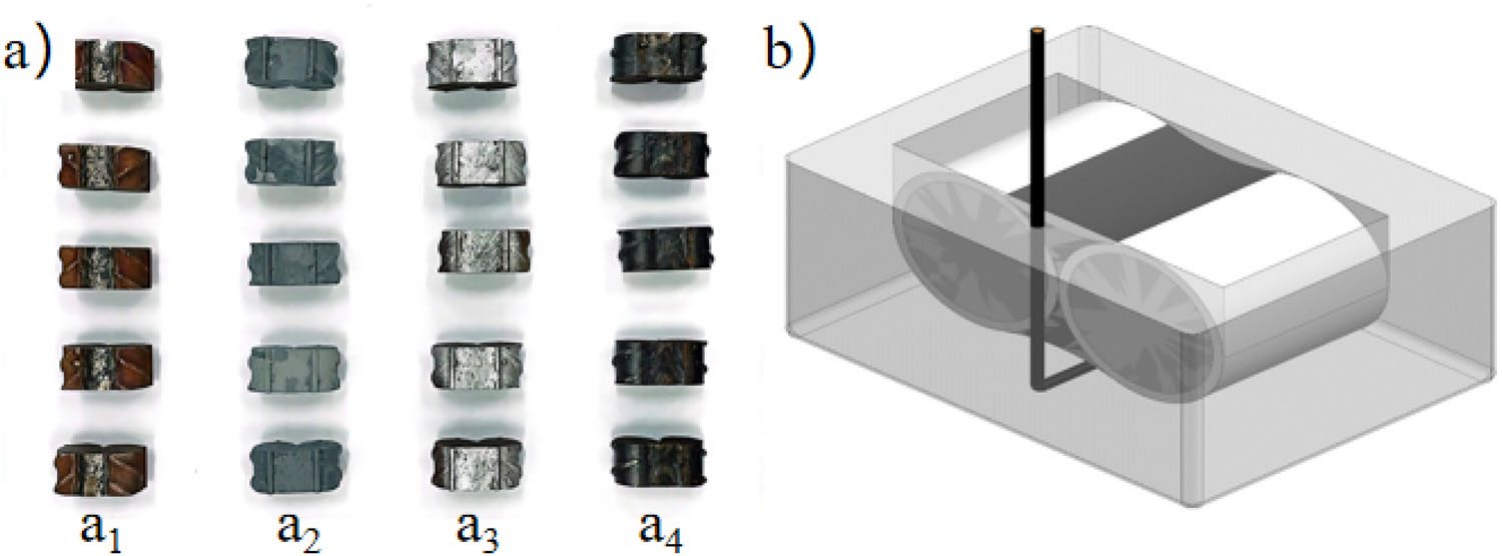
Electrochemical samples preparation: (**a**_1_) CRW samples, (**a**_2_) ZCRW samples, (**a**_3_) PCRW samples, (**a**_4_) CSW samples, (**b**) Electrochemical sample preparation.

For electrochemical testing, samples from stainless-steel-clad rebar welding (CRW), carbon-steel welding (CSW), painting the clad rebar after welding (PCRW), and cold zinc spraying after clad rebars welding(ZCRW) samples were cut to a length of 16 mm (as shown in Fig. 5a). One side of the the steel bar was covered with plasticine or blue butyl glue, and a wire, insulated, was affoxed to the center of the specimen’s back. Epoxy resin was poured, submerging the section entirely. After curing for 24 h at 20–30 °C, the specimen was removed, cleaned of the covering plasticine or blue butyl glue, covered it was cleaned, as depicted in Fig. 5b.

### Experimental equipment and procedures

The electrochemical corrosion test was conducted using a Reference3000 & Interface 1010E with IMPS & IMPV electrochemical workstation. A platinum electrode served as the auxiliary electrode, while the reference electrode was a saturated calomel electrode. The initial applied potential is − 0.25 V, moreover, the applied final potential is 0.25 V, the scan rate is 2 mV/s, the sample period is 1 s, and the sample area is 5.12 cm^2^. The time of initial display is 60 s. The solution was selected as 3.5% neutral NaCl solution and the test temperature was 30 ± 1 °C. After the sample is prepared according to Fig. 2b, the wire can be connected to the working electrode for electrochemical testing. The JXA-8230 electron probe microanalyzer facilitated line scans and surface scans to investigate element diffusion and distribution in similar/dissimilar metal welded joints. Firstly, the sample was fixed on the tray with conductive adhesive and the device was vacuumed before test, the probe was used to analyze the element diffusion in the marked area at an accelerated voltage of 15 kV. The BH200M optical microscope was employed for observing the microstructure morphology in the joint area post-welding. The sample section was polished with 180#, 400#, 800# and 1600# sandpaper and metallographic polishing cloth, respectively. The corrosion solution was 4% nitric acid solution. The mixed reagent of HNO_3_:HCL:H_2_O = 1:3:4 was selected to corrode the stainless steel side. The Wyko NT9100 optical surface profile instrument was used to scan the surface corrosion morphology of corroded samples, and the measuring range was 3 mm × 3 mm. The test results were selected by splice of 12 images, and the experimental system threshold was 2%, Force Indensity was 50%, and scan speed was 1X. And other parameters were default. Additionally, a JSM-IT300LA scanning electron microscope (SEM) was utilized for studying corrosion morphology.

## Results and discussion

### Metallographic structure of welded joints

**Figure 6:**
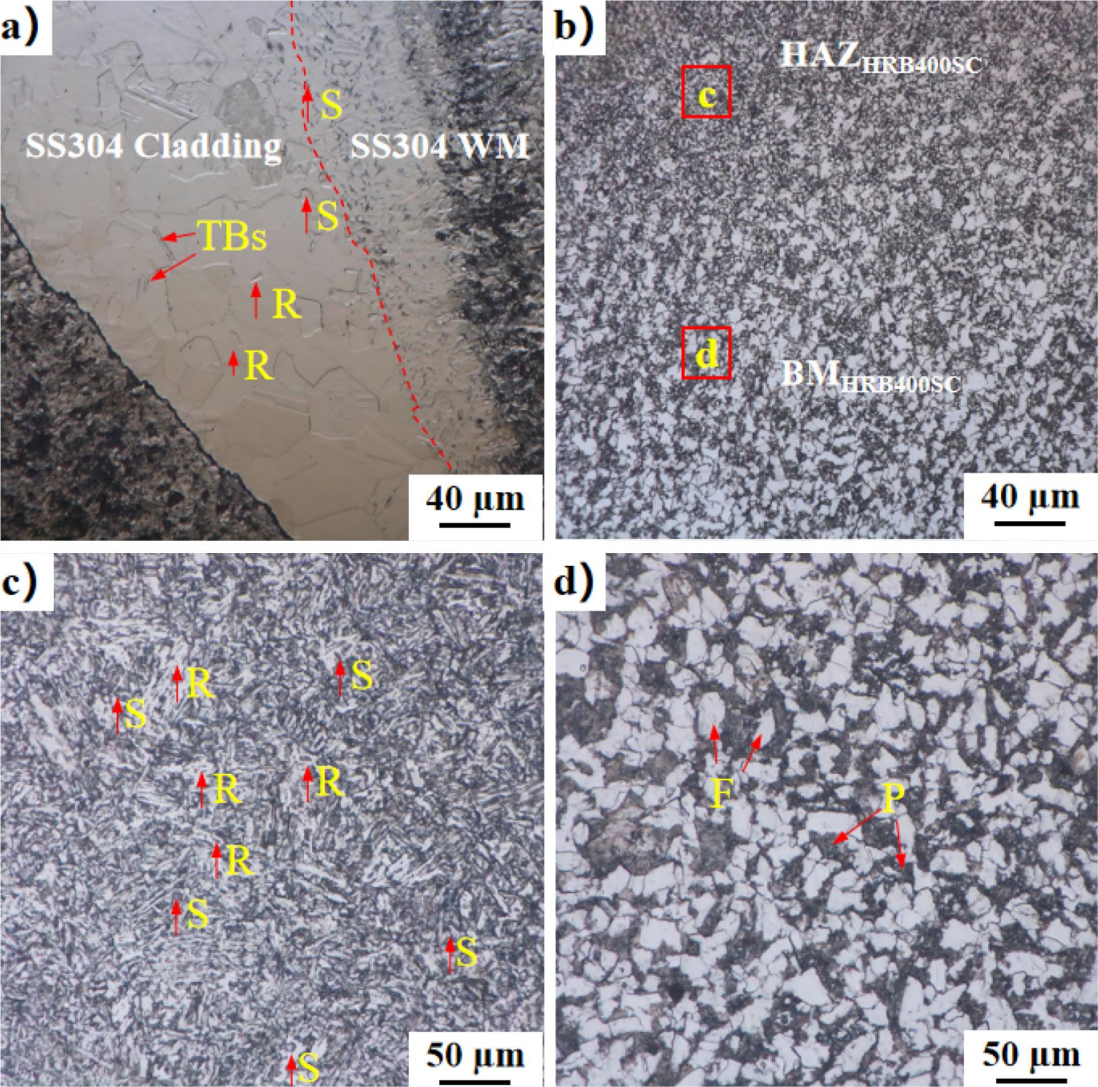
Metallographic microstructure in different regions of welded joints of clad rebar: (**a**) HAZ of SS304, (**b**) HAZ and matrix transition zone(BM) of HRB400CS, (**c**) HAZ of HRB400, and (**d**) BM of HRB400.

As illustrated in Fig. 4b, the welding process of the clad steel bar resulted in welding penetration. The dissimilar metal welded joint emerged from the combination of stainless-steel welding material and the carbon-steel matrix, while the stainless-steel cladding of the clad steel bar formed a same-metal welded joint. In Fig. 6a, between the HRB400 carbon-steel matrix and the welding material, the stainless-steel cladding reveals coarse austenite grains with numerous twin boundaries (TBs) and high-angle grain boundaries (HAGBs). This observation aligns with findings by Zhiguo et al.^20^ in their study of nuclear austenitic stainless steel. Qing et al.^21^ discovered the formation of TBs by the austenite body in the ∑ phase during austenite morphism. In addition, SS304 grains near the weld fusion line exhibit refinement due to dynamic recrystallization (DRX) under thermal coupling. This DRX includes both continuous dynamic recrystallization (CDRX) and discontinuous dynamic recrystallization (DDRX)^22^. The presence of fine recrystallized grains inside deformed grains (Arrow R) suggests CDRX in SS304, while the proportion of TBs decreases and HAGBs increase in the heat-affected zone (HAZ) compared with the SS304 base metal. This transformation is attributed to strain-induced grain boundary migration, converting TBs into HAGBs. Simultaneously, SS304 exhibits a bow appearance of HAGBs (Arrow S), indicating DDRX. Because TBs facilitate nucleation for DDRX and the SS304 layer fault energy is low, twin-induced dynamic recrystallization (TDRX) occurs, but CDRX does not. Sabzi et al.^23^ discovered that DRX caused by deformed nanotwins are activated during laser cladding when preparing austenitic stainless steel. Thus, DDRX and TDRX are the primary mechanisms of grain refinement in SS304^24,25^.

Figure 6b displays the HAZ of HRB400 and matrix transition zones of HRB400. The microstructure of HAZ of HRB400 consists of ferrite, bainite and pearlite, with pearlite distributed along the ferritic grain boundary. As shown in Fig. 6c, the bainite appears as granular and black needle, and the ferrite includes massive ferrite, grain boundary ferrite, and acicular ferrite. Wang et al.^26,27^ welded SS304 and Q235 mild steel through friction stir welding and observed that acicular ferrite and pearlite were produced in the HAZ of Q235. The ferrite grains were refined, while the pearlitic grains were coarse. Notably, small HAGBs grains in the plastic deformation crystals of the HRB400 HAZ (Arrow R) indicate CDRX in this region. Additionally, the HAGBs arch image is more pronounced in the HAZ region (Arrow S). Some HAGBs migrate into adjacent grains, leading to the formation of new grains at grain boundaries. Consequently, in the HAZ of HRB400 region, the main mechanisms for grain refinement are CDRX and DDRX. Figure 6d illustrates that the carbon-steel matrix microstructure comprises ferrite and pearlite^24,25^.

### Line and face scanning results of EPMA

**Figure 7:**
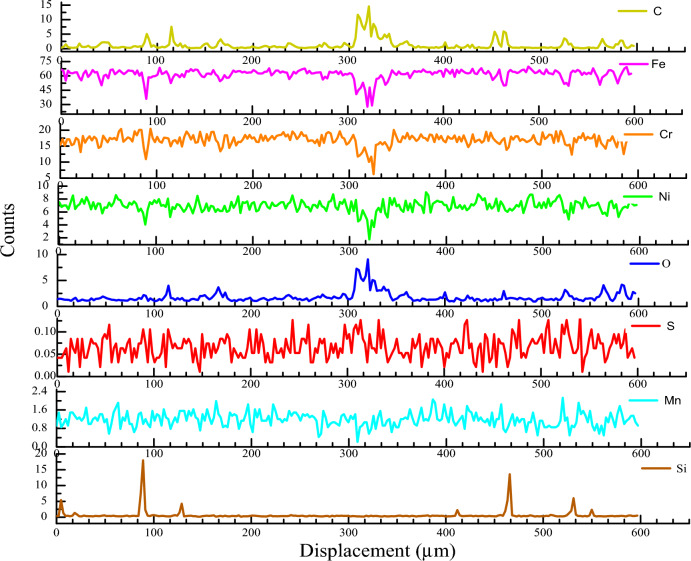
Line scanning of SS cladding and fusion zone of SS304 WM.

The EPMA sample, as shown in Fig. 4b, was subjected to line scanning at the position of Line 1 to detect element diffusion. The results of the line scan are depicted in Fig. 7.

According to the EPMA line scan results, C, Fe, Cr, Ni, and other elements diffused at the fusion line position, with O peaking in the HAZ of fusion between the stainless-steel cladding and the welding material. This indicates the presence of oxides near the fusion line, suggesting exposure to air during the welding process. The diffusion distances are as follows: C (32.76 µm), Fe (37.44 µm), Cr (21.06 µm), Ni (19.06 µm), and O (39.78 µm). The peak of C at 308–340 µm results from the high energy of HAZ in the heating process, the austenitic stainless-steel base metal is in a saturated state, and carbide (M_23_C_6_) and nitride (Cr_2_N) precipitate along the grain boundaries with cooling and solidification. During the initial precipitation solidification of austenite, part of γ-Fe transforms into α-Fe and Fe_3_C by eutectoid reaction, increasing the C content. The concentrations of Ni and Cr decrease at 308–327 µm and 306–327 µm, attributable to the welding melting zone. Austenitic stainless steel is affected by welding heat, generating a welding molten pool during solidification, with austenitic phase as the initial precipitated phase. Ni is an austenitic forming element and results in dilution. Cr forms ferrite during the transition from the gamma phase to the alpha phase. Liu et al.^28^ welded four types of austenitic stainless steels by MAG welding and noted that the welds of the four kinds of welded joints were solidified in the austenite-ferrite mode, and the δ-phase ferrite was distributed in the austenite grains. Consequently, the concentrations of Ni and Cr successively reduce, followed by ferrite formation when the welding temperature is lower than the solid phase line. Near the fusion boundary, the Cr_eq_/Ni_eq_ ratio reaches 2:1 to 3:1, accelerating ferrite formation along the HAZ austenite grain boundary. The formed ferrite limits grain growth and reduces the sensitivity of HAZ to liquefaction cracks^29^.

**Figure 8:**
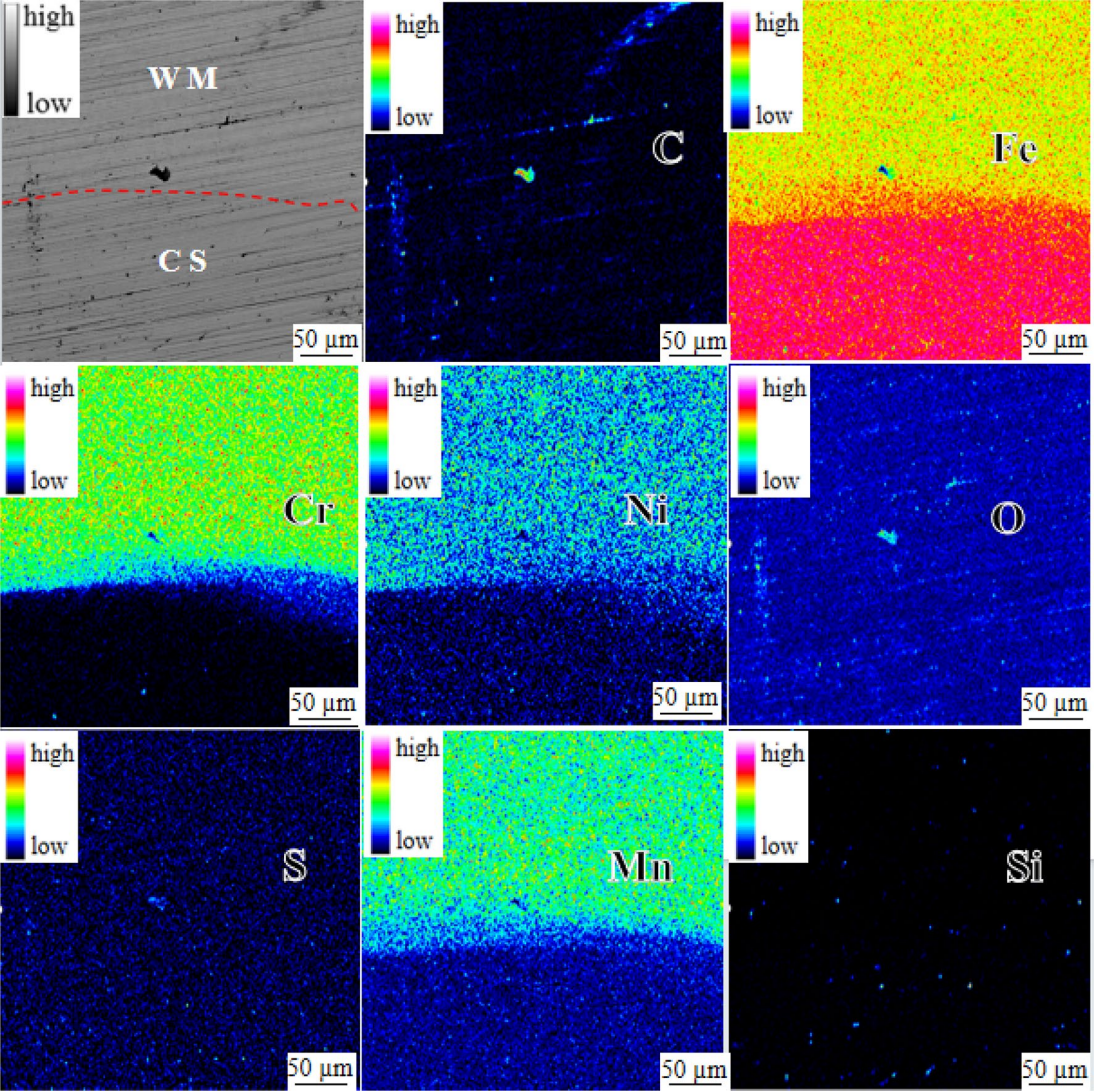
Facing scanning of fusion zone between SS304 welding material and HRB400 matrix of clad rebar

As depicted in Fig. 4b, element distribution was detected via face scanning at the position of Map 2. The face scanning results are presented in Fig. 8. The red line indicates the fusion boundary between the SS304 welding material and the clad rebar matrix. According to the EPMA results, Fe, Cr, Ni, Mn, and other elements exhibit diffusion, with Fe having a diffusion distance of 117.32 µm, Cr (58.66 µm), Ni (58.66 µm), and Mn (25.14 µm). Notably, the black substance exhibits high concentrations of C, O, and S and low concentrations of Fe, Cr, Mn, and Si, indicating welding slag inclusion, a welding defect.

### Electrochemical corrosion test

**Figure 9:**
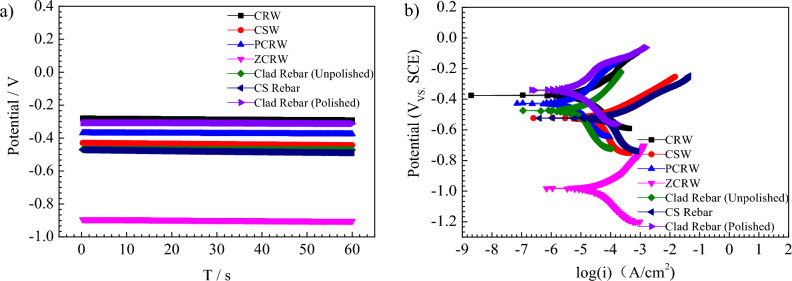
Potential curves of different welded joints in 3.5% NaCl solution: (**a**) Open circuit potential (OCP), and (**b**) Potentiodynamic polarization (PD).

The influence of welding connections on the corrosion resistance of clad rebars was examined through open circuit potential and potentiodynamic polarization curve tests using various samples, including CRW, CSW, PCRW, and ZCRW, polished clad rebars, carbon-steel bars, and unpolished clad rebars. The open circuit potential results are depicted in Fig. 9a, with a test duration of 60 s. Figure 9b illustrates the results of potentiodynamic polarization curves for each sample in a 3.5% NaCl solution.

As illustrated in Fig. 9a, polished clad rebars and CRW exhibit the highest potential, followed by PCRW. Carbon-steel bars, welded using stainless-steel welding wire, exhibit a potential similar to unpolished clad rebars. The lowest potential is observed in ZCRW. This suggests that carbon-steel and zinc coatings have a greater thermodynamic tendency for anodic dissolution. Since the thermodynamic criterion of corrosion tendency is the potential difference, moreover, the larger thermodynamic tendency of anodic dissolution refers to the process of anodic dissolution under a certain external potential when the metal is anode, the greater the thermodynamic tendency, the more likely it is to corrode, the smaller the corrosion potential or the greater the corrosion current. It can be seen from Fig. 6b, carbon-steel and zinc coatings have the lowest corrosion potential, so they have a greater thermodynamic tendency for anodic dissolution. In Fig. 9b, the polarization curves of polished clad rebars indicate passivation between − 3.4 and − 4.2 A/cm^2^, while other joints show limited passivation. The polarization curves for CRW, PCRW, polished and unpolished clad rebars are similar in shape, indicating a shared corrosion mechanism where the cathode-oxygen reaction is controlled by activation. The cathodic polarization slope of CSW, carbon-steel bars, and zinc coating is greater than the anode, signifying that the corrosion process is governed by oxygen reduction diffusion. The clad rebar exhibits the highest corrosion potential after polishing, with an E_corr_ of − 335 mV, followed by: CRW, PCRW, clad rebar (unpolished), and CSW, carbon-steel bar, with corrosion potential E_corr_ values of − 338 mV, − 393 mV, − 460 mV, − 518 mV, and − 523 mV, respectively. The ZCRW exhibited the lowest self-corrosion potential, with E_corr_ = − 931 mV. Thus, in 3.5% NaCl solution, the corrosion resistance of the polished clad rebar is the highest, followed by CRW, PCRW, clad rebar (unpolished), CSW, and carbon-steel. Sohail et al.^30^ utilized the same method to obtain the electrochemical corrosion parameters such as :corrosion potential, corrosion current density, and the Tafel constants. The ZCRW is susceptible to corrosion in the solution because zinc exhibits active chemical properties and participates in chemical reactions with the external environment, generating zinc oxides and other compounds on the coated steel surface. This result is in agreement with the accelerated test, and the samples can be ranked as follows: clad steel bar (polished) > CRW > PCRW > CSW > clad rebar (unpolished) > carbon-steel bar > ZCRW.

As indicated by the data in Table 1, it is evident that the minimum icorr is observed in PCRW and polished clad rebars, measuring 9.7 × 10^–9^ A/cm^2^ and 1.41 × 10^–8^ A/cm^2^, indicating that these two samples exhibit the slowest reaction rate with the solution, the lowest activity, and the highest corrosion resistance. Following closely are CRW, unpolished clad rebars, CSW, carbon-steel bars, and ZCRW, with corresponding self-corrosion current densities of 2.08 × 10^–8^, 3.45 × 10^–8^, 1.11 × 10^–7^, 1.32 × 10^–7^, and 4.76 × 10^–7^ A/cm^2^. The slowest corrosion rate is observed in PCRW, with a corrosion rate (CR) of 4.26 mm/a, similar to polished clad rebars (6.18 mm/a). Following this, CRW, unpolished clad rebars, CSW, carbon-steel bars, and ZCRW exhibit CRs of 9.14, 15.16, 48.95, 58.13, and 209.30 mm/a, respectively. The i_corr_ of unpolished clad rebars is 3.45 × 10^–8^ A/cm^2^, while the corrosion current density of polished clad rebars is 1.41 × 10^–8^ A/cm^2^. The self-corroded current density of polished clad rebar is 2.08 × 10^–8^ A/cm^2^. I_corr_ indicates the property of corrosion resistance, the little of the i_corr_, the higher is the corrosion resistance. Thus, the corrosion resistance of CRW is 39.6% higher than that of unpolished clad rebars, 48.2% lower than polished clad rebars, 81.3% higher than CSW, and 84.2% higher than carbon-steel bars. In a marine service environment, the reaction speed with seawater, from slow to fast, is observed as PCRW < clad rebar (polished) < CRW < clad rebar (unpolished) < CSW < carbon-steel bar < ZCRW.

**Table 1:** Potentiodynamic polarization curve parameters of the joints.

Samples	E_corr_/(mV)	i_corr_/(A/cm^2^)	Βa/ (mV/decade)	Βc/ (mV/decade)	CR(Tafel)/ (mm/a)
CRW	− 338	2.08 × 10^–8^	156.3	160.7	9.14
CSW	− 518	1.11 × 10^–7^	100.7	338.1	48.95
PCRW	− 393	9.7 × 10^–9^	224.5	300.8	4.26
ZCRW	− 931	4.76 × 10^–7^	247.6	1 × 10^15^	209.30
Clad rebar (unpolished)	− 460	3.45 × 10^–8^	523.9	649.1	15.16
Carbon steel bar	− 523	1.32 × 10^–7^	92.9	265.9	58.13
Clad rebar (polished)	− 335	1.41 × 10^–8^	106.7	155.3	6.18

After the OCP and potentiodynamic polarization experiments, we carried out the accelerated test on the surface of the sample for 600 s to observe four samples of corrosion morphology. Given that the potentiodynamic polarization curve is solely used to detect the self-corrosion of the sample surface coating in reaction with the solution, and the cold spraying of zinc after welding of the clad steel bar belongs to the cathodic protection method of sacrificial anode, the corrosion damage degree of the sample surface cannot be observed^31^. Therefore, the corrosion current is set as 0.8 A, corrosion time as 600 s, and an electrochemical acceleration experiment was carried out to detect the corrosion morphology and surface roughness after corrosion of four kinds of welding joints. This further characterizes the corrosion influence of coating welding, carbon-steel welding, clad rebar welding with painting, and clad rebar welding with zinc spraying, providing insights into the corrosion resistance of these welding joints.

**Figure 10:**
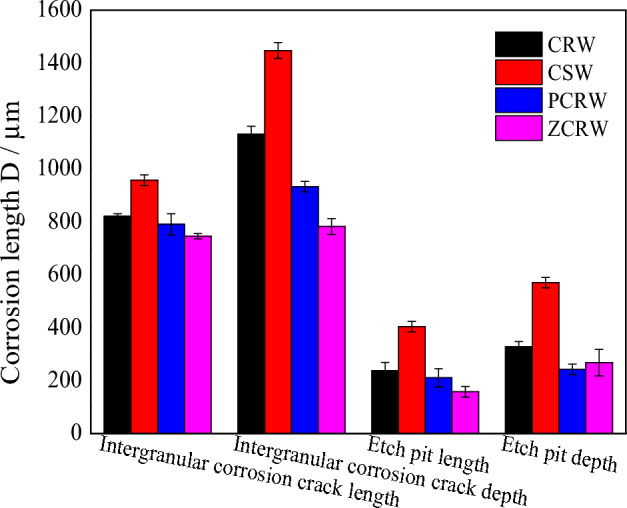
Morphology statistics of different samples under corrosion times of 600 s.

Figure 10 presents the statistics of the corrosion morphologies of various samples over a 600 s corrosion period. Notably, the damage caused by corrosion in ZCRW is the least severe, while carbon-steel bars exhibit the most pronounced corrosion morphology. A passivation film forms to hinder further reactions between the solution and the coating, thereby safeguarding the clad rebar. In carbon-steel, the welding pitting pits are the largest in terms of both shape and number, and the intergranular corrosion cracks boast the greatest length and depth, indicative of inferior corrosion resistance. After welding, the paint detaches, exposing the clad steel bar matrix to NaCl solution, playing a role in the initial stages of corrosion. Subsequently, the corrosion morphology resembles that of the clad rebar.

### The observation on corrosion morphology

**Figure 11:**
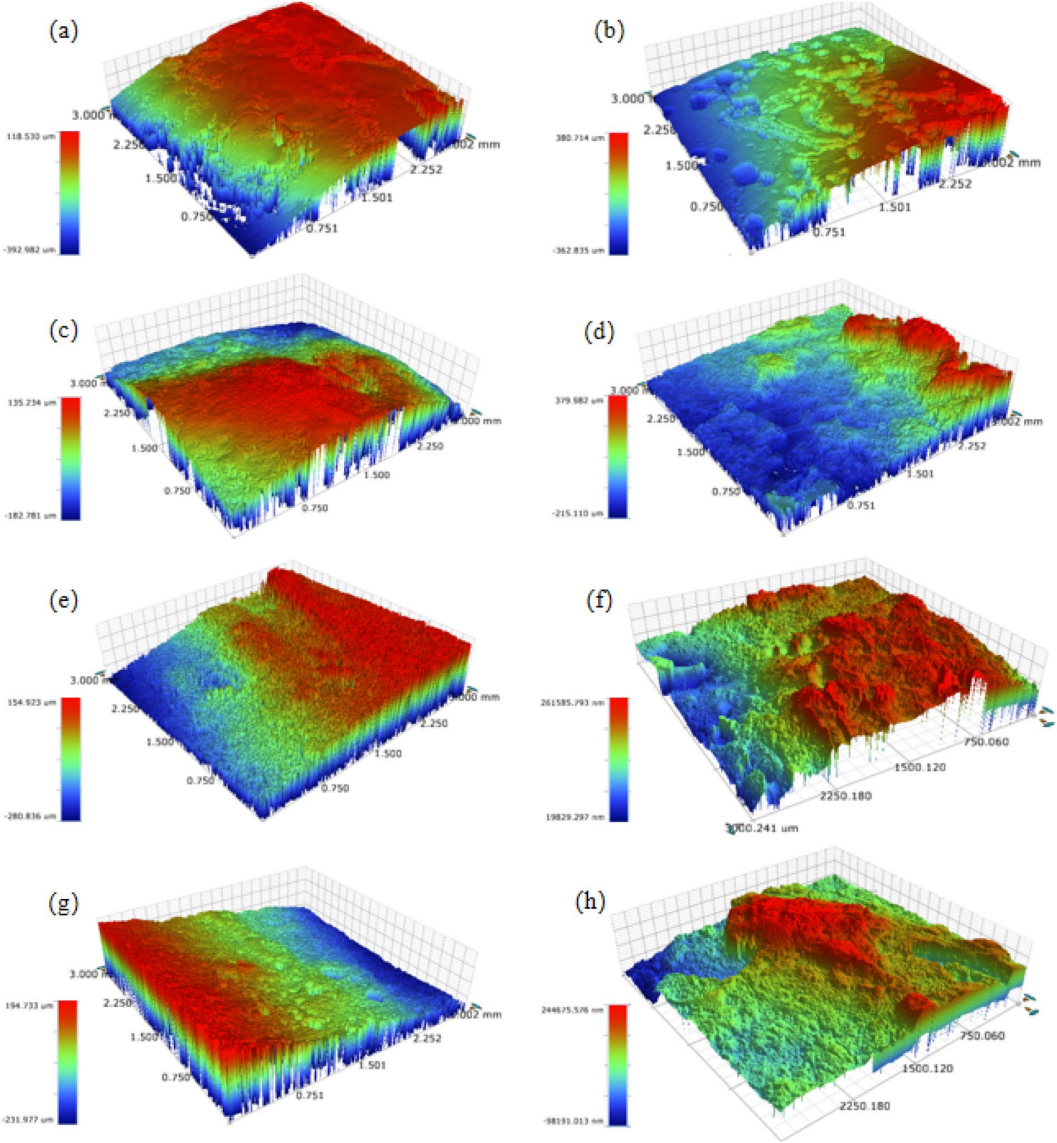
Surface corrosion morphology of four welded joints: (**a**) Before corrosion test of the CRW, Ra = 48.9 µm; (**b**) Corrosion test of the CRW for 600 s, Ra = 131.4 µm; (**c**) Before corrosion test of the CSW, Ra = 43.3 µm; (**d**) Corrosion test of the CSW for 600 s, Ra = 179.6 µm; (**e**) Before corrosion test of the PCRW, Ra = 78.0 µm; (**f**) Corrosion test of the PCRW for 600 s, Ra = 155.5 µm; (**g**) Before corrosion test of the ZCRW, Ra = 93.0 µm; (**h**) Corrosion test of the ZCRW for 600 s, Ra = 104.2 µm;

In order to compare the corrosion degree of different welded joints quantitatively, Wyko NT9100 optical surface profiler was utilized to observe the surface corrosion morphology of different welded joints before and after corrosion test, and the surface roughness (Ra) of the observation area was tested, as shown in Fig. 11.

The changes of Ra values before and after corrosion of welded joints in increasing order are as follows: ZCRW,11.2 µm, PCRW, CRW and the CSW,which the surface roughness change value are 11.2 µm, 77.5 µm, 82.5 µm and 136.3 µm. This is consistent with the results of OM experiment, indicates that the clad rebar welded joints with zinc has the best surface protection effect and the best corrosion resistance. Omid et al.^32^ presented a novel low-cost method based on dynamic speckle pattern analysis for real-time monitoring of pit initiation and growth in a two-point bending specimen.

**Figure 12:**
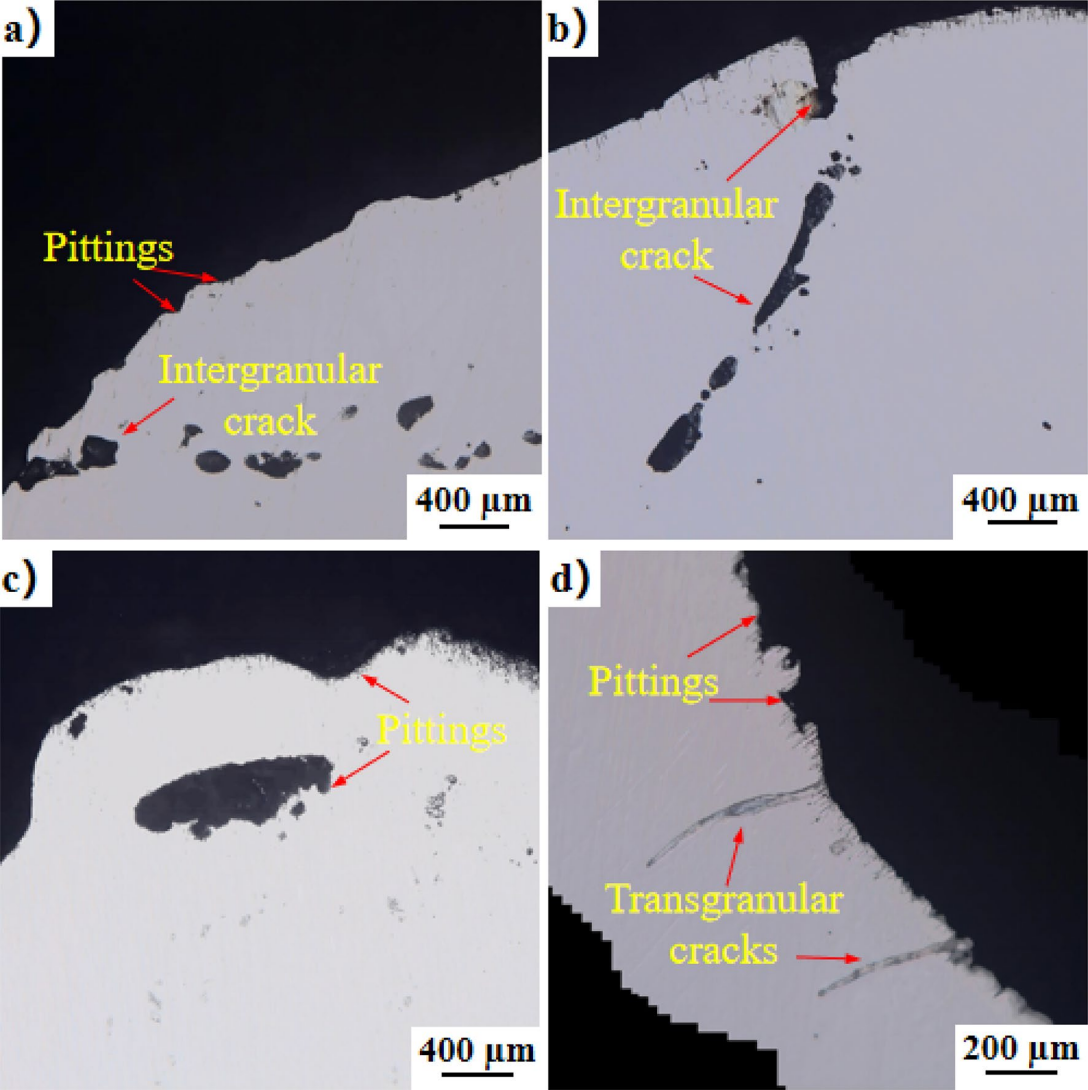
Corrosion pit topography of different welding samples under corrosion for 600 s: (**a**) CRW corrosion for 600 s, (**b**) CSW corrosion for 600 s, (**c**) PCRW corrosion for 600 s, and (**d**) ZCRW corrosion for 600 s.

As illustrated in Fig. 12, after 600 s of welding corrosion, distinct pitting pits and intergranular corrosion emerge, with a width ranging from 96 to 380 µm and a depth of 32–240 µm. The intergranular corrosion exhibits a length of 320 µm and a depth of 112 µm. Corrosion in the carbon-steel matrix is concentrated in the fusion zone between the SS304 cladding and SS304 WM, as well as between the SS304 WM and HRB400 matrix. The corrosion zone forms along the longitudinal edge of the stainless-steel cladding. Pitting corrosion is the predominant form of damage, accompanied by transgranular corrosion with a depth of approximately 309.1–498.2 µm and a transgranular corrosion depth of 290.9 µm. The highest corrosion rate is along the composite surface,recorded depth at 424.1 µm. After 600 s of corrosion in PCRW, numerous pitting pits appear in the stainless-steel cladding, and the carbon-steel matrix corrodes along the composite interface with a corrosion length of about 300.0 µm, indicating the initiation of paint detachment, bringing the steel bar and welding surface into contact with the reaction medium. In the case of zinc spraying corrosion after 600 s, corrosion pits manifest in the stainless-steel welding material, and the carbon-steel matrix of the steel bar also begins to corrode along the composite interface. Intergranular corrosion cracks also appear in the stainless-steel coating.

### The corrosion mechanism of SS304

**Figure 13:**
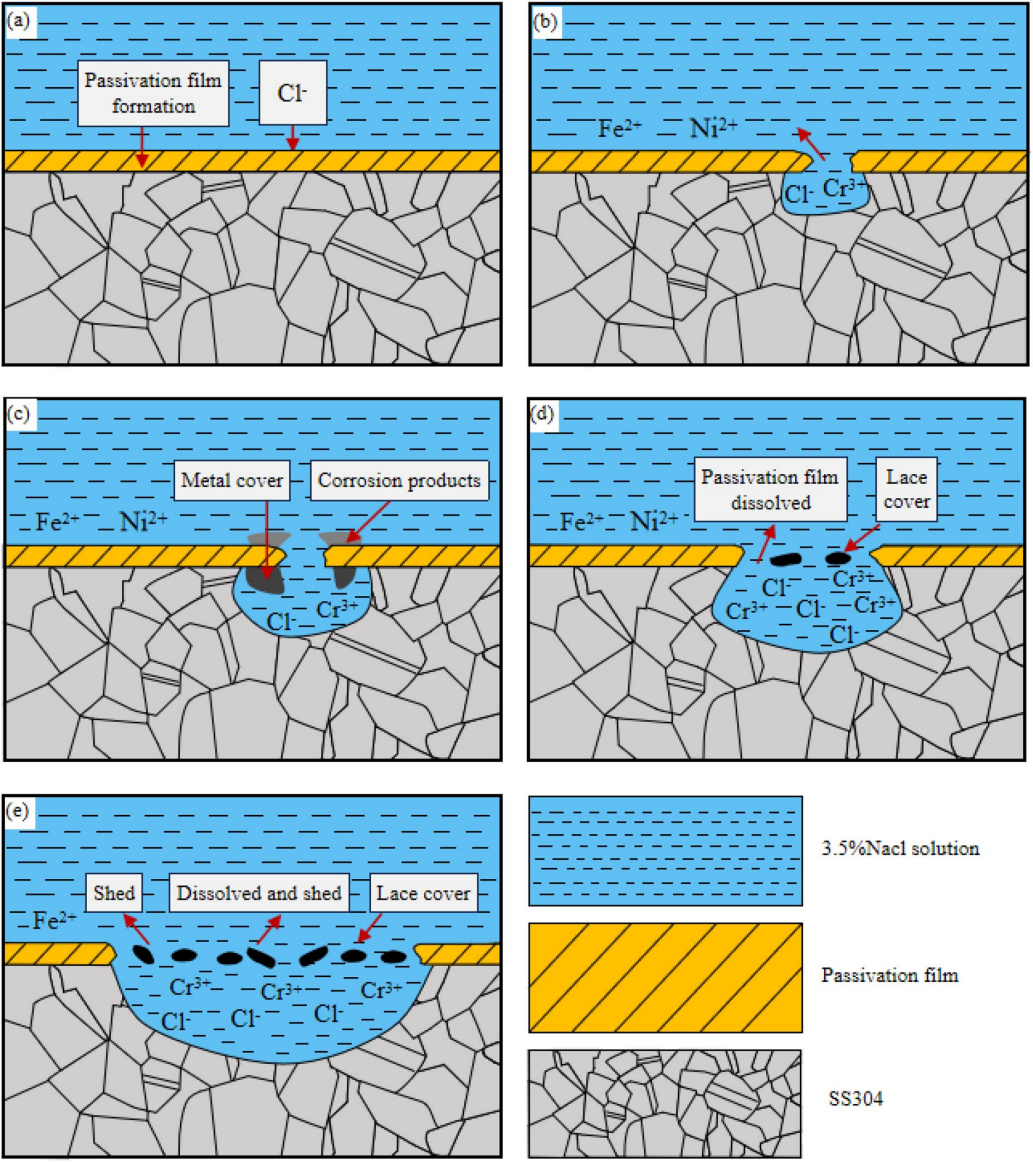
SS304 tissue corrosion process in 3.5% NaCl solution.

The corrosion process of SS304 is shown in the Fig. 13 At the initial stage of corrosion, passivation film was formed on the surface of SS304, and solution rich in Cl^-^ is easily adsorbed by the surface defect of passivation film, and the Cl^−^ penetrating the surface film to the interface between metal, moreover,it causes local damage to the film, resulting in pitting initiation, as shown in Fig. 13a. The metal dissolution occurs at the bottom of pitted pits, producing metal ions (Fe^2+^, Cr^3+^, Ni^2+^, etc.) and diffusing. As the concentration of metal ions increased, the hydrolyzed H^+^ increased, resulting in auto catalytic dissolution, which improves the activity of H^+^ and accelerates the dissolution at the bottom of the pit, as shown in Fig. 13b. As plenty of metal ions were produced, the corrosion products generated and covered at the pit, forming a closed area in the pit, and the oxygen concentration in the pit is lower than outside, moreover, an was formed, further accelerates the dissolution of active substances in the pitting pit, as shown in Fig. 13c. In the further development of pitting, the higher ion concentration in the pit than outside, the metal cover plate formed above the pitting pit began to dissolve, and re-passivation occurred in the pitting pit, forming a lace-like morphology, as shown in Fig. 13d. Sun et al.^32^ found that the anode metal salt concentration must be greater than the critical line C*, which is 75% to 80% of the saturated chloride concentration C_sat_, to maintain high-spenged dissolution, the corrosion occurs below the critical line, and the passivation appears above the critical line. The porous lace-cap pitting produced outside the pitting pit. Shao et al.^33^ indicated that the Cl^−^ and Br^−^ exist simultaneously, the Cl^−^ are not prone to metastable corrosion, moreover, it could inhibit the growth of corrosion pits in the seawater environment. When the passivation zone is insufficient to maintain the lace cover, it will shed or dissolve, as shown in Fig. 13e.

## Conclusions

1. The welding of clad rebars results in the formation of homologous/dissimilar metal welded joints, with distinct types of DRX occurring at various positions within the joints. This results in various forms of grain refinement in both the HAZ of stainless steel and carbon steel. Notably, SS304 experiences grain refinement primarily through DDRX and TDRX, while in HRB400CS, CDRX and DDRX dominate as the main mechanisms of grain refinement in the HAZ.
2. Electrochemical tests revealed that the clad rebar (polished) exhibited the smallest self-corrosion current and the highest corrosion resistance. After welding, the corrosion resistance of the clad steel bar surpassed that of the unpolished clad steel bar by 39.6%, exceeds the carbon-steel bar by 81.3%, and outperformed the carbon-steel bar by 84.2%. Consequently, the clad steel bar retained commendable corrosion resistance after undergoing the welding process. The corrosion process and mechanism of SS cladding was analyzed.
3. Among the different welded components, the surface corrosion damage was the least pronounced in the ZCRW. The zinc coating on the welded steel bar preferentially engaged in a reaction with the solution, resulting in the attachment of zinc oxide to the sample’s surface. Consequently, the corrosion potential increased, playing a pivotal role in safeguarding the sample’s surface. Given the enhanced corrosion resistance of the clad rebar after polishing, coupled with the durability of the zinc coating, the coated steel bar must be polished after welding, followed by the application of a zinc spray to the surface.

## Data Availability

The datasets generated and/or analysed during the current study are available from the corresponding author on reasonable request.

## References

[1] Dong J, Han E, Ke W (2007). Introduction to atmospheric corrosion research in China. Sci. Technol. Adv. Mater..

[2] Yuan J, Ou Z (2021). Research progress and engineering applications of stainless steel- reinforced concrete structures. Adv. Civil Eng..

[3] Han EH, Wang J (2023). Effect of surface state on corrosion and stress corrosion for nuclear materials. Acta Metall. Sin..

[4] Wang BJ, Luan JY, Xu DK (2019). Research progress on the corrosion behavior of magnesium–lithium-based alloys: A review. Acta Metall. Sin. (Engl. Lett.).

[5] Hou BR (2014). Corrosion protection technology of ocean steel structure spray splash zone. Progr. Mater. China.

[6] Hou, B. R. Corrosion hazard of steel structure in spray splash area under Marine environment. *Sci. Chin. *(11), 21–22.

[7] Wang H, Wang K, Wang W, Huang L, Peng P, Yu H (2019). Microstructure and mechanical properties of dissimilar friction stir welded type 304 austenitic stainless steel to Q235 low carbon steel. Mater. Charact..

[8] Cui S, Shi Y, Cui Y (2019). The influence of microstructure and chromium nitride precipitations on the mechanical and intergranular corrosion properties of K-TIG weld metals. Constr. Build. Mater..

[9] Wei P, Mingfang Wu, Liu D (2022). Electrochemical corrosion behavior of MIG-welded 7N01-T4 aluminum alloy by ER5356 and ER5087 welding wires. Materials.

[10] Sohail MG (2020). Electrochemical behavior of mild and corrosion resistant concrete reinforcing steels. Constr. Build. Mater..

[11] Shi Y, Cui S, Zhu T, Gu S, Shen X (2018). Microstructure and intergranular corrosion behavior of HAZ in DP-TIG welded DSS joints. J. Mater. Process. Technol..

[12] Jegdić B, Bobić B, Radojković B, Alić B, Radovanović L (2019). Corrosion resistance of welded joints of X5CrNi18-10 stainless steel. J. Mater. Process. Technol..

[13] Yang Z, Huang H (2023). Corrosion behavior of ADC12 aluminum alloy welded joint using tungsten inert gas welding in 3.5wt.% NaCl solution. Mater. Chem. Phys..

[14] Senthilkumar S, Manivannan S (2023). Influence of heat input on the mechanical characteristics, corrosion and microstructure of ASTM A36 steel welded by GTAW technique. Heliyon.

[15] Li J, Li H, Liang Y (2020). Effects of heat input and cooling rate during welding on intergranular corrosion behavior of high nitrogen austenitic stainless steel welded joints. Corros. Sci..

[16] Gucwa M, Winczek J, Giza K (2019). the effect of welding methods on the corrosion resistance of 304 stainless steel joints. Acta Phys. Polon. A.

[17] Sriba A, Vogt JB (2021). Galvanic coupling effect on pitting corrosion of 316L austenitic stainless steel welded joints. Met. Mater. Int..

[18] Li X, Shao Y, Miao W (2020). Galvanic corrosion behaviors of the low-carbon ferritic stainless steel ERW (electrical resistance welding) joint in the simulated seawater. Anti-Corros. Methods Mater..

[19] Li Z, Qian XH, Tan JP (2022). Profile change law of clad rebars and the formation mechanism of composite interfaces during hot rolling. Materials.

[20] Zhiguo W (2022). Effect of twin-related boundaries distribution on carbide precipitation and intergranular corrosion behavior in nuclear-grade higher carbon austenitic stainless steel. Corros. Sci..

[21] Zhou Q, Liu J, Gao Y (2020). The heritage of the twin microstructure in the sigma phase formed from deformed austenite. J. Alloys Compd..

[22] Kaiming Z (2022). Dynamic recrystallization behavior and numerical simulation of S280 ultra-high strength stainless steel. J. Mater. Res. Technol..

[23] Eskandari SH (2022). Deformation twinning-induced dynamic recrystallization during laser powder bed fusion. Scr. Mater..

[24] Logé RE (2022). Nanotwinning-assisted recrystallization. Nat. Mater..

[25] Tiamiyu AA (2022). Nanotwinning-assisted dynamic recrystallization at high strains and strain rates. Nat. Mater..

[26] Wang H (2019). Microstructure and mechanical properties of dissimilar friction stir welded type 304 austenitic stainless steel to Q235 low carbon steel. Mater. Charact..

[27] Yu WX, Liu BX, Chen CX (2020). Microstructure and mechanical properties of stainless steel clad plate welding joints by different welding processes. Sci. Technol. Weld. Join..

[28] Bahremand F, Shahrabi T, Ramezanzadeh B (2023). Sustainable development of an effective anti-corrosion film over the St12-steel surface against seawater attacks using Ce(III) ions/tri-sodium phosphate anions. Sci. Rep..

[29] Pedram O, Jamali R, Abbasian V (2023). Evaluation of pitting corrosion by dynamic speckle pattern analysis. Sci. Rep..

[30] Sohail MG (2021). Electrochemical corrosion parameters for active and passive reinforcing steel in carbonated and sound concrete. Mater. Corros..

[31] Frankel GS (1998). Pitting corrosion of metals: A review of the critical factors. J. Electrochem. Soc..

[32] Sun DM, Jiang YM (2009). Stainless steel pitted lacing cover falls off with stable growth. J. Zhengzhou Univ..

[33] Shao Z, Yu D, Shao D (2024). A protective role of Cl^−^ ion in corrosion of stainless steel. Corros. Sci..

