# Author Correction: Welding-induced corrosion and protective measures for clad rebars in neutral chloride environments

**DOI:** 10.1038/s41598-024-66137-3

**Published:** 2024-07-09

**Authors:** Zecheng Zhuang, Weiping Lu, Lei Zeng, Jianping Tan, Xuehai Qian, Zhen Li, Wei Jiang, Yong Xiang

**Affiliations:** 1 Light Alloys Research Institute, Central South University, Changsha, 410083 China; 2 Guangxi Normal University of Science and Technology, LaiBin, 546199 China; 3 State Key Laboratory of Precision Manufacturing for Extreme Service Performance, Central South University, Changsha, 410083 China; 4 School of Mechanical and Electrical Engineering, Central South University, Changsha, 410083 China; 5 Technology Centre, Guangxi Liuzhou Iron and Steel Group Ltd., Liuzhou, 545002 China

Correction to: *Scientific Reports* 10.1038/s41598-024-56348-z, published online 22 May 2024

The Funding section in this Article was incomplete.

“This research was financially supported by the key R & D project of Guangxi Zhuang Autonomous Region (2021AB16016), the Science and Technology Plan Project of Liu Zhou City (2021AA0101B001), and the University Research Basic Ability Improvement Project of Guangxi Zhuang Autonomous Region (2020KY23018), and Guangxi Science and Technology Base and Talent Project (2022AC20024).”

now reads:

“This research was financially supported by the key R & D project of Guangxi Zhuang Autonomous Region (2021AB16016), the Science and Technology Plan Project of Liu Zhou City (2021AA0101B001), and the University Research Basic Ability Improvement Project of Guangxi Zhuang Autonomous Region (2020KY23018), Guangxi Science and Technology Base and Talent Project (2022AC20024), and Guangxi Science and Technology Normal University Key Laboratory project “Automotive Intelligent Manufacturing and Robotics Key Laboratory” (GXKSKYPT2021008).”

The original Article has been corrected.

